# Increase in Beta-Band Activity during Preparation for Overt Speech in Patients with Parkinson’s Disease

**DOI:** 10.3389/fnhum.2017.00371

**Published:** 2017-07-24

**Authors:** Peter Sörös, Nuria Doñamayor, Catharina Wittke, Mohamed Al-Khaled, Norbert Brüggemann, Thomas F. Münte

**Affiliations:** ^1^Department of Neurology, University of Lübeck Lübeck, Germany; ^2^Psychiatry and Psychotherapy, School of Medicine and Health Sciences, University Hospital Karl-Jaspers-Klinik, University of Oldenburg Oldenburg, Germany; ^3^Neuroimaging Unit, University of Oldenburg Oldenburg, Germany; ^4^Research Center Neurosensory Science, University of Oldenburg Oldenburg, Germany; ^5^Department of Psychiatry, University of Cambridge Cambridge, United Kingdom; ^6^Institute of Psychology II, University of Lübeck Lübeck, Germany

**Keywords:** Parkinson’s disease, speech production, brain rhythms, β-band, EEG

## Abstract

Speech impairment is a frequent and often serious symptom of Parkinson’s disease (PD), characterized by a disorder of phonation, articulation and prosody. While research on the pathogenesis of the prominent limb motor symptoms has made considerable progress in recent years, the pathophysiology of PD speech impairment is still incompletely understood. To investigate the neural correlates of speech production in PD, EEG was recorded in 14 non-demented patients with idiopathic PD and preserved verbal fluency on regular dopaminergic medication (8 women; mean age ± SD: 69.5 ± 8.0 years). The control group consisted of 15 healthy age-matched individuals (7 women; age: 69.7 ± 7.0 years). All participants performed a visually-cued, overt speech production task; required utterances were *papapa* and *pataka*. During the preparatory phase of speech production, in a time window of 200–400 ms after presentation of the visual cue, β-power was significantly increased in PD patients compared to healthy controls. Previous research has shown that the physiological decrease of β-power preceding limb movement onset is delayed and smaller in PD patients off medication and normalizes under dopaminergic treatment. By contrast, our study demonstrates that β-power during preparation for speech production is higher in patients on dopaminergic therapy than controls. Thus, our results suggest that the mechanisms that regulate β-activity preceding limb movement and speech production differ in PD. The pathophysiological role of this increase in β-power during speech preparation needs to be determined.

## Introduction

Impairment of speech production, first described by James Parkinson in his classical Essay on the Shaking Palsy (Parkinson, 1817), is an often serious sequela of Parkinson’s disease (PD), characterized by a disorder of phonation, articulation and prosody (Sapir, 2014). During the course of the illness, a majority of patients experiences difficulties of speech production. At the baseline visit of a large cohort study including 419 PD patients, 51% of patients reported at least slight impairment of speech production (Perez-Lloret et al., 2012). In a group of 125 patients with PD, speech intelligibility of PD patients as rated by listeners unfamiliar with dysarthric speech was significantly worse compared to unaffected age-matched controls (Miller et al., 2007). With progression of the disorder, speech impairment increases in frequency and intensity (Skodda et al., 2013). In the early stage of the disease, a disorder of phonation is often the leading symptom of PD speech impairment, presenting with a breathy and harsh voice of reduced loudness (Ho et al., 1998; Holmes et al., 2000). In a later stage, difficulties of articulation may develop. The articulatory precision in the production of consonants—in particular, stop consonants such as /k/, /p/ and /t/—is typically reduced (Ackermann and Ziegler, 1991) and speech rate declines (Martínez-Sánchez et al., 2016). Prosody, the natural variations in loudness, pitch and rhythm of fluent speech, is frequently impaired as well (Darkins et al., 1988).

PD is an adult-onset neurodegenerative disorder associated, at its core, with a loss of dopaminergic neurons within the substantia nigra that project to the striatum and, hence, changes of the functional connectivity within basal ganglia-thalamocortical circuits (Wichmann et al., 2011; Göttlich et al., 2013). Multiple lines of evidence suggest that neural oscillations within these circuits differ between healthy individuals and patients with PD (Oswal et al., 2013; Brittain and Brown, 2014; Brittain et al., 2014). Subcortical recordings of local field potentials, mainly from the subthalamic nucleus, identified excessive neural oscillations in the β-band in PD patients (Bronte-Stewart et al., 2009; Hirschmann et al., 2011). This increase in rhythmic brain activity in basal ganglia-cortical circuits is now regarded as a key concept in the pathophysiology of motor and cognitive deficits in PD (Oswal et al., 2013). Several studies have shown that task-related modulation of cortical β-band activity is reduced in PD patients compared with healthy controls (Pollok et al., 2012; Heinrichs-Graham et al., 2014; te Woerd et al., 2014).

While research on the pathogenesis of the prominent limb motor symptoms in PD has made considerable progress (Wichmann et al., 2011), the pathophysiology of PD speech impairment is still little understood (Sapir, 2014). PD speech impairment has traditionally been characterized as a pure speech motor disorder—a hypokinetic dysarthria—due to hypokinesia, bradykinesia and rigidity of laryngeal and orofacial muscles. This pathophysiological model has been challenged by several experimental and therapeutic observations, though. Studies of orofacial muscle tone in patients with PD suggested that rigidity alone does not fully explain the characteristics of PD speech disorder (Sapir, 2014). Moreover, dopaminergic stimulation with L-dopa or apomorphine, highly effective in the treatment of limb motor symptoms in PD, does not consistently improve PD speech disorder (Kompoliti et al., 2000; Schulz and Grant, 2000; Ho et al., 2008). Finally, deep brain stimulation of the subthalamic nucleus may even result in a deterioration of PD speech disorder (Skodda et al., 2014; Tsuboi et al., 2015).

The aim of the present study was to investigate β-band rhythmic activity in the preparatory phase of overt non-lexical speech production in PD patients and in a control group of age-matched healthy individuals using EEG. We chose two target utterances, *papapa* and *pataka*. Both trisyllabic utterances are challenging because they require fast alternating (diadochokinetic) motions of the articulators. *Pataka* is particularly difficult because the three stop consonants demand a swift and precise movement of the tongue from the front to the back of the oral cavity. Actual speaking is associated with electromyographic activity in many different muscles, including the temporalis muscle that covers large parts of the temporal and parietal bone (Tuller et al., 1981). To minimize contamination of our EEG recordings by muscle artifacts, we only chose to analyze the preparatory phase, between the onset of the visual cue and the onset of vocalization.

## Materials and Methods

### Participants

**Figure 1:**
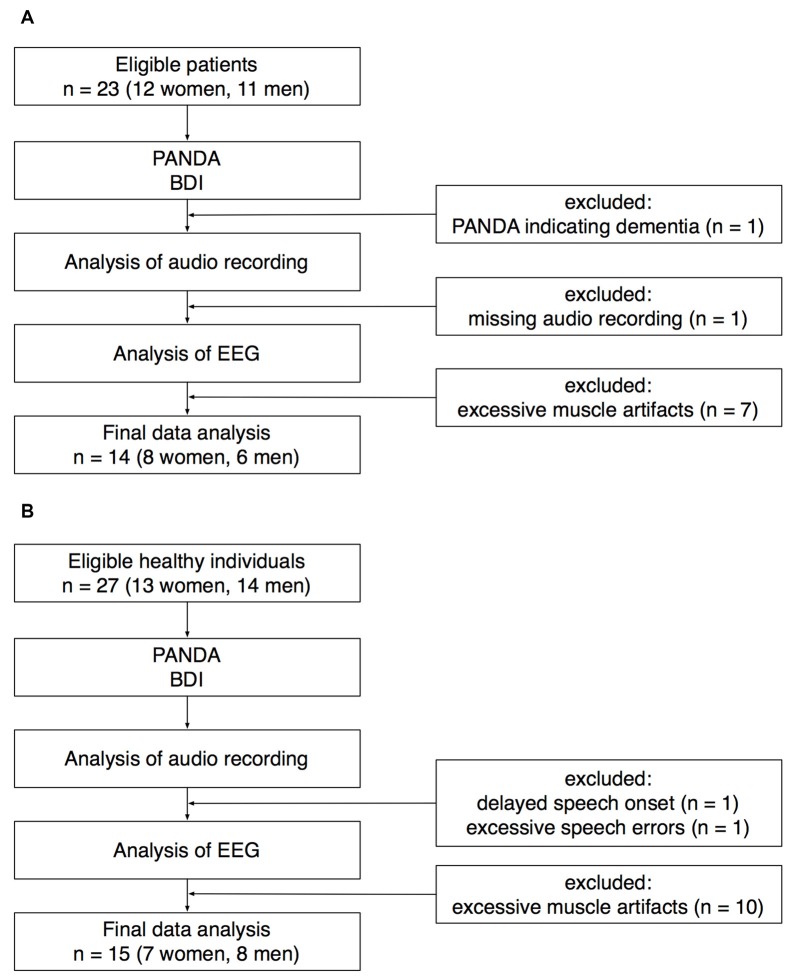
Recruitment of patients **(A)** and healthy controls **(B)**. PANDA, Parkinson Neuropsychometric Dementia Assessment. BDI, Beck Depression Inventory.

Twenty-three patients with idiopathic PD (12 women) were recruited at the Outpatient Clinic, Department of Neurology, University of Lübeck. For details of recruitment, see Figure 1. One patient was excluded because the Parkinson Neuropsychometric Dementia Assessment (PANDA, Kalbe et al., 2008) indicated cognitive impairment. EEG recordings from seven patients had to be excluded because of excessive muscle activity during preparation for speaking. Another patient was excluded because the audio recording was missing due to a technical failure. The data sets of the remaining 14 patients (8 women) were analyzed for this study. Mean age ± standard deviation was 69.5 ± 8.0 years (age range: 52–81 years).

All patients were diagnosed by an experienced neurologist; the initial diagnosis was made between 3 years and 16 years before this study was performed. All patients received antiparkinsonian medication at the time of the study (mean equivalent L-dopa dose: 677 mg/day). To assess the severity of motor symptoms, part III of the Unified Parkinson’s Disease Rating Scale (UPDRS) was performed in all patients (Fahn and Elton, 1987). The mean UPDRS III score ± SD was 17 ± 11 (minimum: 3, maximum: 44).

Twenty-seven healthy individuals (13 women) were recruited for an age-matched control group. Twenty-five participants were recruited through the subject database of the Department of Neurology, two participants were healthy spouses of the patients recruited for this study. EEG recordings from 10 healthy participants had to be excluded because of excessive muscle activity during preparation for speaking. One healthy participant was excluded because of an exceptionally delayed speech onset. In this participant, the time between the onset of the visual cue and the onset of speaking was on average 1965 ms (for the rest of the control group, speech latency was 659 ± 153 ms). Another healthy participant was excluded because of an exceptionally high number of incorrect speech responses (72%). The data sets of the remaining 15 healthy participants (7 women) were analyzed for this study. Mean age ± SD was 69.7 ± 7.0 years (age range: 55–80 years).

All included patients and control participants were right-handed, had normal or corrected-to-normal vision and no history of neurological or psychiatric disorder (except PD in the patient group). The five cognitive tasks of the PANDA (Kalbe et al., 2008) showed no cognitive impairment. The Beck Depression Inventory (Beck et al., 1961) indicated no depressive symptoms. To assess verbal fluency at the behavioral level, all participants were asked to produce as many words beginning with “m” as possible within 2 min as part of the Regensburger Wortflüssigkeits-Test (Aschenbrenner et al., 2000).

This study was carried out in accordance with the recommendations of the Research Ethics Board of the University of Lübeck with written informed consent from all subjects. All subjects gave written informed consent in accordance with the Declaration of Helsinki. The protocol was approved by the REB. Participants received a remuneration of 40 Euro.

### Experimental Paradigm

A visually cued overt speech production task was performed during the EEG recording. Participants were seated in a comfortable chair with their eyes about 80 cm in front of a 20 inch LCD computer screen. To cue speech production, the syllables *papapa* or *pataka* were shown in the middle of the screen for 2000 ms. Patients were instructed to speak aloud the required utterance as soon as the cue appears. The onset-to-onset time interval between cues was 5000 ms. To reduce task-switching effects and to facilitate the task, a blocked presentation was chosen. Each block was preceded by the instruction “Jetzt kommt *papapa* (Now we’ll present *papapa*)” or “Jetzt kommt *pataka* (Now we’ll present *pataka*)”. Each block consisted of five *papapa* and five *pataka* trials. Five blocks were presented in pseudo-randomized order for each condition. In total, 25 papapa and 25 pataka trials were recorded. All stimuli were presented using Presentation software (Neurobehavioral Systems, Berkeley, CA, USA)^1^.

All utterances were recorded using a microphone and stored for off-line analysis with the open source audio software Audacity^2^. All utterances were examined by the same listener (CW) and double-checked by another researcher (PS). Trials without a correct, intelligible utterance were excluded from further data analysis. In the patient group, on average 0.9 trials per participant were excluded because a correct verbal response was missing (minimum: 0 trials, maximum: 5 trials). In the control group, on average 1.7 trials per participant were excluded due to a missing correct response (minimum: 0 trials, maximum: 9 trials). Speech latency (the time difference between onset of the instruction and onset of overt speech) was determined for all correct responses and all participants. On an individual basis, speech latencies were *z*-transformed and trials with exceptionally short or long latencies (*z* < −3 or *z* > 3) excluded. On average, 0.9 trials per participant in the patient group (minimum: 0, maximum: 2) and 0.7 trials per participant in the control group (minimum: 0, maximum: 2) were excluded after *z-transformation* of latencies. The average individual speech latency was finally calculated for every participant.

### EEG Acquisition

EEG data were recorded in a sound-dampened and electromagnetically-shielded room using an elastic 32 electrode cap (Electro-Cap International, Eaton, OH, USA)^3^ and a Synamps amplifier (Compumedics Neuroscan, Singen, Germany)^4^. To record brain electric activity, 29 tin electrodes were mounted on the scalp according to the 10/20 system with modified combinatorial nomenclature (American Electroencephalographic Society, 1994). Scalp electrodes were referenced online against the left mastoid. To monitor vertical and horizontal eye movements, electrodes were placed above and below the left eye and on the outer canthus of each eye. Data was sampled at 250 Hz, with a bandpass filter of 0.01–50 Hz. Electrode impedances were kept below 5 kΩ.

### EEG Analysis

Raw data were re-referenced to the average activity of both mastoid electrodes. An independent component analysis (ICA) was performed using an extended information maximization algorithm (Bell and Sejnowski, 1995) as implemented in EEGLAB (Delorme and Makeig, 2004)^5^ to identify and remove components related to eye blinks and ocular movements (Hoffmann and Falkenstein, 2008).

**Figure 2:**
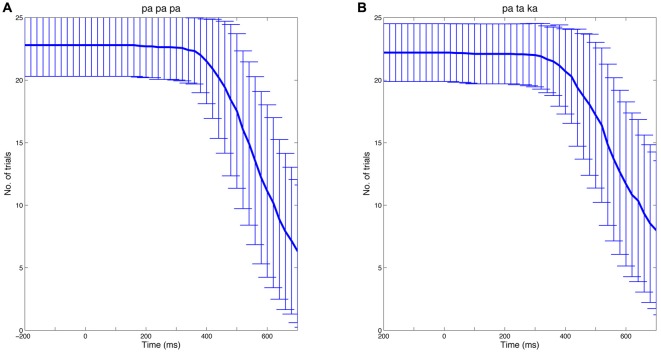
Number of trials over time. The analysis of EEG data focused on the preparatory phase of speech production. Thus, individual EEG epochs were truncated at speech onset. The Figure shows the mean number of trials at a given time (bold blue line) for the *papapa* condition **(A)** and the *pataka* condition **(B)** across all participants. The error bars represent the standard deviation.

To calculate time-frequency spectra, single-trial data were convolved with a complex Morlet wavelet using the open source MATLAB toolbox FieldTrip (Oostenveld et al., 2011)^6^. The width of the wavelet was set at seven cycles. To avoid contamination of EEG data by speech-related muscle artifacts, individual EEG epochs were truncated at speech onset. Thus, the number of analyzed trials and hence the signal-to-noise ratio decreased after approximately 400 ms. Figure 2 illustrates the number of trials (mean ± SD) available at a given time point for the *papapa* and the *pataka* conditions, separately.

After wavelet transform, oscillatory power in the studied frequencies (1–50 Hz, linear increase) was computed for each trial in the time window −1000 ms to speech onset and averaged for each subject time-locked to the onset of the visual cue before calculating a grand average. The interval −100 ms to 0 ms (onset of visual cue) served as baseline for all computations. A baseline close to the onset of the visual cue was chosen to minimize contamination by muscle artifacts related to the preceding trial.

After inspection of the time course of β-activity (Figure 7), we decided to test changes in β-power in the time window of 200–400 ms after onset of the visual cue.

**Figure 3:**
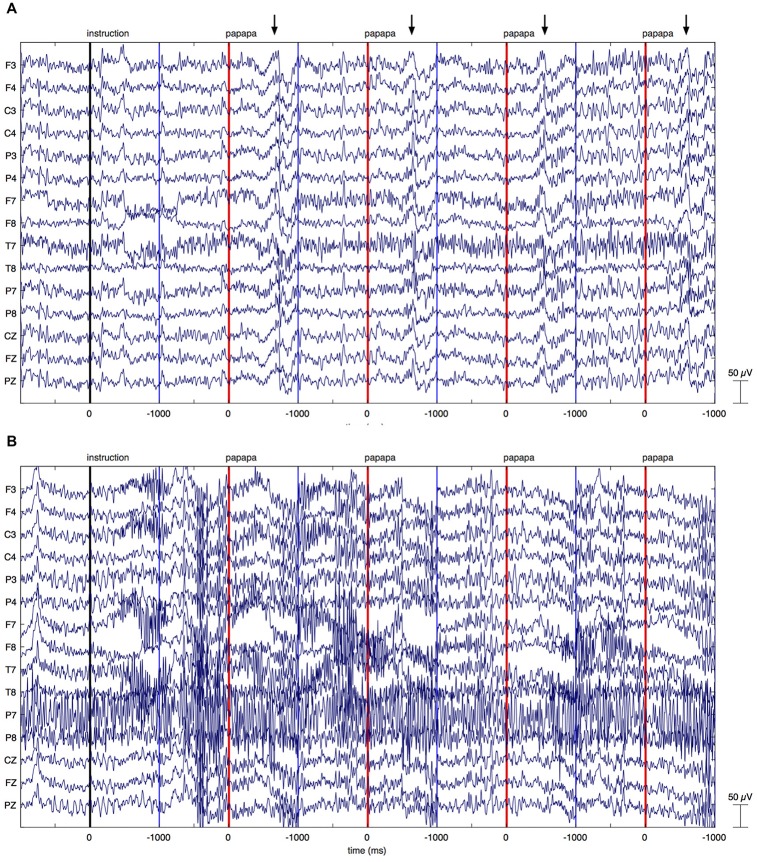
Individual EEG data. The figure illustrates EEG recordings of two patients with Parkinson’s disease (PD), showing the first four *papapa* trials of the experiment. The onset of the visual cue (*papapa*) is represented by a red line. Epochs of EEG data have been extracted between −1000 and 1000 ms relative to the onset of the cue. The upper recording **(A)** displays artifacts related to overt speech, starting around 500 ms after onset of the cue (marked by arrows). As these artifacts started with speech onset, the recording was included in the final data analysis. The lower recording of another patient with PD **(B)** shows massive high-frequency artifacts due to muscular activity. This recording was excluded from the final data analysis.

**Figure 4:**
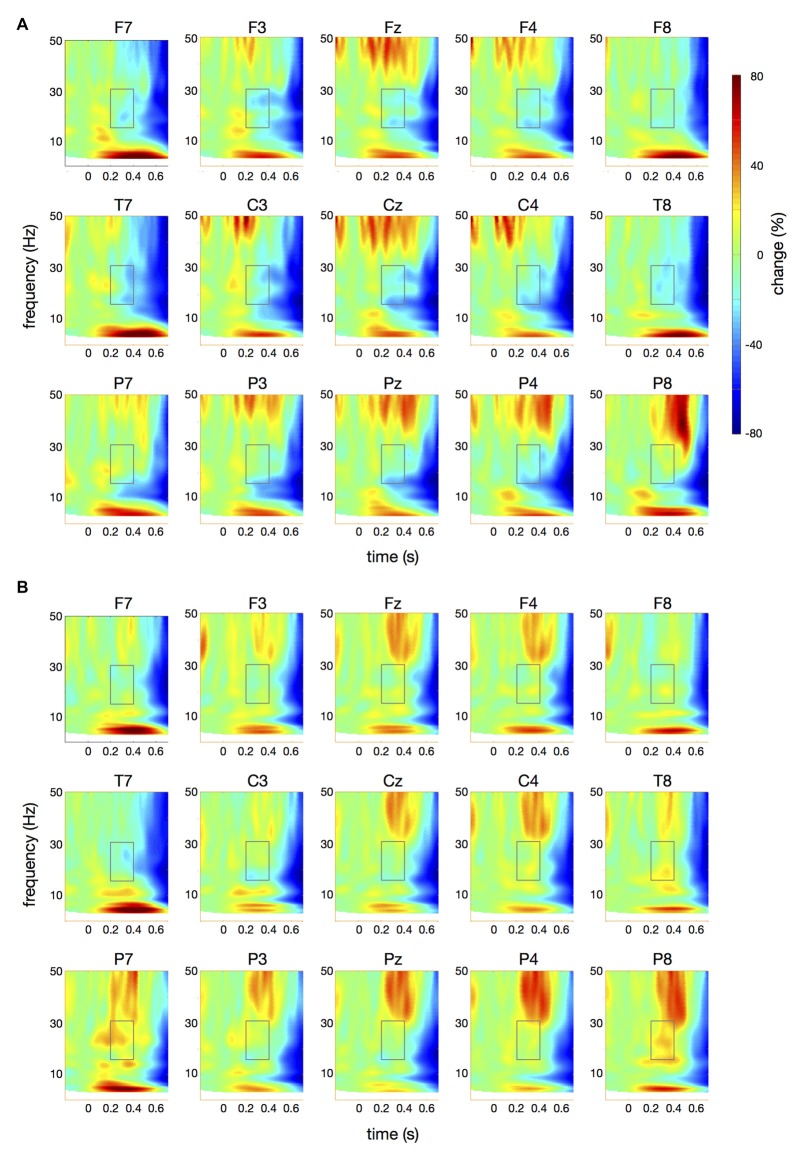
Time-frequency spectra for the *papapa* condition. The figure represents averaged changes in spectral power relative to a baseline period of −100 ms to 0 ms during the preparation for speech production. Time (in seconds) is shown on the *x*-axis; 0 s is the onset of the visual cue (*papapa*). Frequency (1–50 Hz) is shown on the *y*-axis. Time-frequency spectra are shown for controls **(A)** and patients **(B)**. The boxes represent the frequency range (16–31 Hz) and time window (200–400 ms) of the ANOVA.

**Figure 5:**
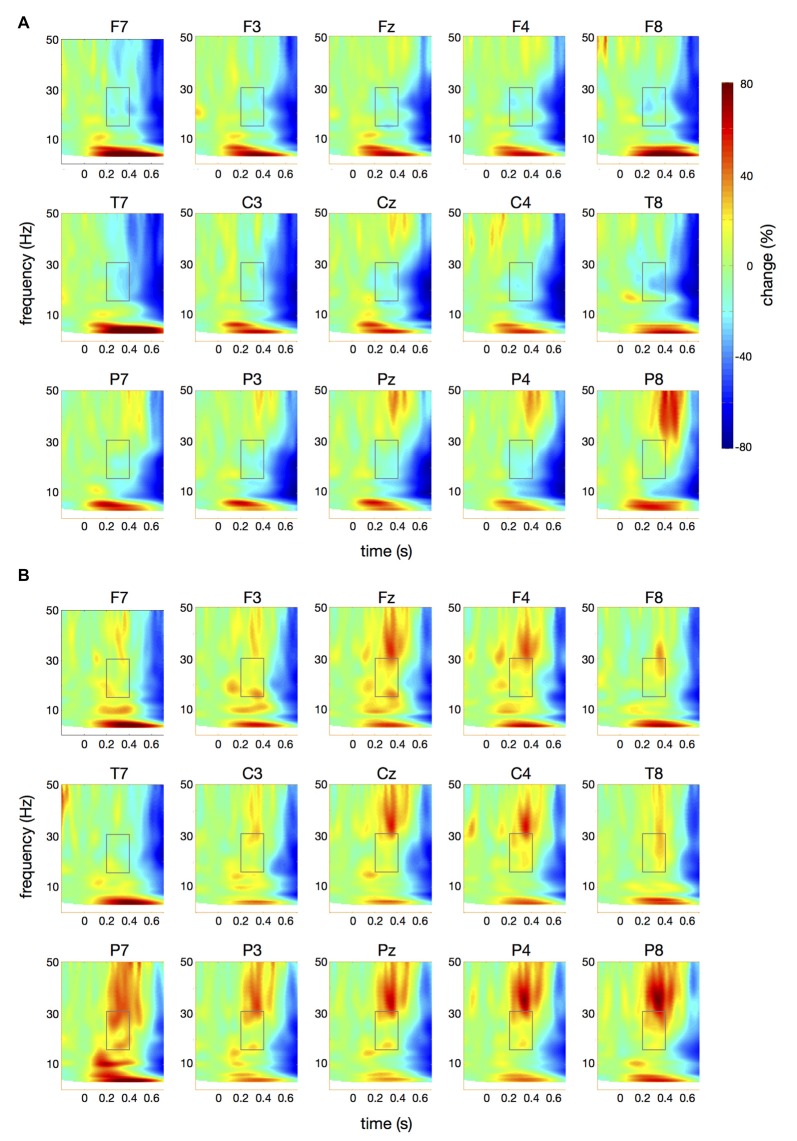
Time-frequency spectra for the *pataka* condition. The figure represents averaged changes in spectral power relative to a baseline period of −100 ms to 0 ms during the preparation for speech production. Time (in seconds) is shown on the *x*-axis; 0 s is the onset of the visual cue (*pataka*). Frequency (1–50 Hz) is shown on the *y*-axis. Time-frequency spectra are shown for controls **(A)** and patients **(B)**. The boxes represent the frequency range (16–31 Hz) and time window (200–400 ms) of the ANOVA.

**Figure 6:**
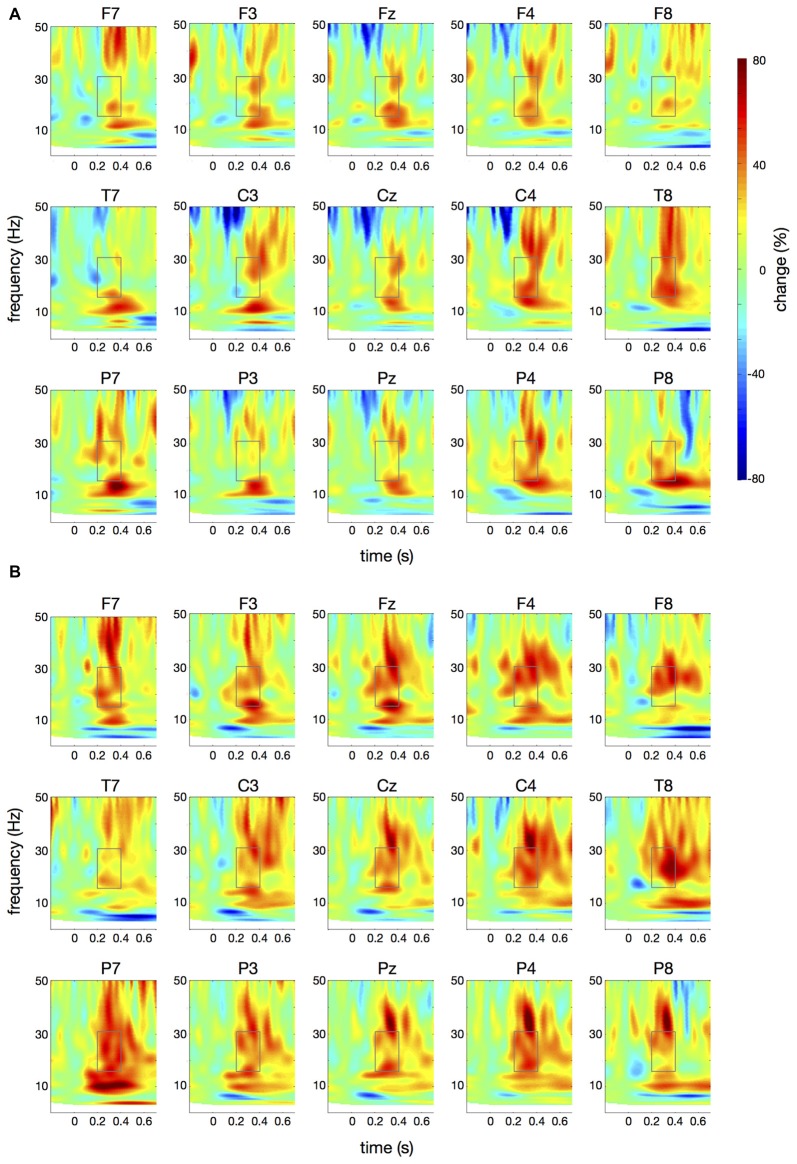
Differences in time-frequency spectra between controls and patients. For this figure, averaged changes in spectral power in controls, relative to a baseline period of −100 ms to 0 ms, were subtracted from changes in spectral power in patients. Yellow and red colors indicate higher spectral power in patients vs. controls. Time (in seconds) is shown on the *x*-axis; 0 s is the onset of the visual cue. Frequency (1–50 Hz) is shown on the *y*-axis. **(A)** displays the difference in spectral power for the *papapa* condition, **(B)** for the *pataka* condition. The boxes represent the frequency range (16–31 Hz) and time window (200–400 ms) of the ANOVA.

**Figure 7:**
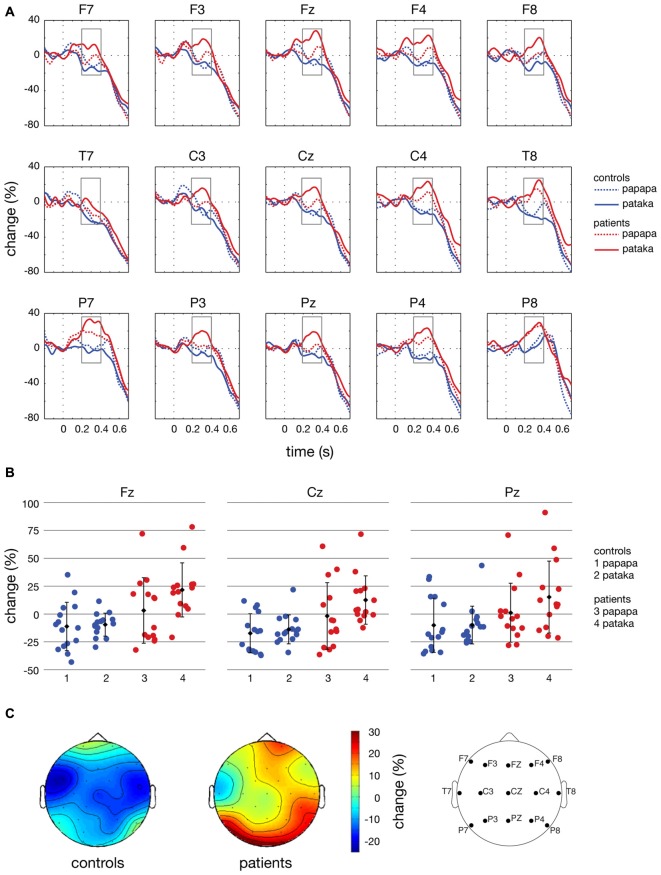
Time courses, individual changes and topographies of spectral power in the β-band. **(A)** shows averaged time courses of spectral power in the β-band (16–31 Hz) relative to a baseline period of −100 to 0 ms for controls (blue lines) and patients (red lines). Dashed lines depict the *papapa* condition, solid lines the *pataka* condition. Time (in seconds) is shown on the *x*-axis; the vertical line at 0 s represents the onset of the visual cue. Relative change (in % of the baseline value) is shown on the *y*-axis. The boxes represent the time window (200–400 ms) of the ANOVA. **(B)** demonstrates the changes in β-band power relative to the baseline for each individual participant, averaged across the time window of 200–400 ms. Healthy controls are shown in blue (1: *papapa* condition; 2: *pataka* condition), patients with PD are shown in red (3: *papapa* condition; 4: *pataka* condition). Data were plotted for the central electrodes Fz, Cz and Pz. The black diamonds represent the group mean, the error bars the standard deviation. **(C)** displays the topographical distribution of changes in spectral power in the β-band relative to the baseline in controls and patients. For each topological plot, the average of the *papapa* and *pataka* condition was calculated.

An ANOVA was calculated for the averaged power in the β-band (16–31 Hz) and the 200–400 ms time window using the between-subjects factor group (patients vs. controls) and the within-subjects factors condition (*papapa* vs. *pataka*), anteriority of EEG electrodes (with 3 levels: F7/F3/Fz/F4/F8; T7/C3/Cz/C4/T8; P7/P3/Pz/P4/P8) and laterality of EEG electrodes (with 5 levels: F7/T7/P7; F3/C3/P3; Fz/Cz/Pz; F4/C4/P4; F8/T8/P8) using IBM SPSS Statistics (version 23)^7^. Greenhouse-Geisser correction was performed when necessary. To estimate the effect size of ANOVA results, partial *η*^2^ was calculated.

## Results

### Behavioral Data

No significant difference between patients and controls regarding the number of words produced in the verbal fluency test (Regensburger Wortflüssigkeits-Test, Aschenbrenner et al., 2000) was found (16 ± 6 vs. 17 ± 4, *p* = 0.69). During the EEG experiment, speech latency of included trials was not significantly different between patients and controls (651 ± 145 ms vs. 659 ± 153 ms; *p* = 0.89).

### EEG Data

Raw EEG data of two PD patients are shown in Figure 3. Figure 3A displays the first four *papapa* trials of a patient whose data were included in the final analysis. By contrast, Figure 3B displays the first four *papapa* trials of another patient whose data were excluded due to massive muscle artifacts.

Figure 4 illustrates the time-frequency spectra for the *papapa* condition in the control (A) and the patient group (B). Figure 5 illustrates the time-frequency spectra for the *pataka* condition in the control (A) and the patient group (B). The time-frequency spectra in Figure 6 show the differences of spectral power between patients and controls for the *papapa* condition (A) and the *pataka* condition (B). Figure 7A depicts the averaged time courses of spectral power in the β-band for both experimental conditions (*papapa* vs. *pataka*) and groups (patients vs. controls). Figure 7B demonstrates individual changes in β-band power (averaged across the time window of 200–400 ms) for patients vs. controls and for both speech production conditions. The topological distribution of changes in β-power in the time window of 200–400 ms relative to the baseline (−100 to 0 ms) for controls and patients is illustrated in Figure 7C.

In the ANOVA, β-power was significantly higher in PD patients than in controls in the time window of 200–400 ms after the visual cue (significant effect of group; *F*_(1,27)_ = 10.90, *p* = 0.003, partial *η*^2^ = 0.288). The main effect of condition and the interactions condition × group, condition × anteriority, condition × group × anteriority, condition × laterality, condition × group × laterality, condition × anteriority × laterality and condition × group × anteriority × laterality were not significant (*p* > 0.05).

## Discussion

The present study demonstrates significant differences in the time course of oscillatory brain activity in the β-band (16–31 Hz) between PD patients and healthy controls during the preparation for overt speech. In a time window of 200–400 ms after presentation of the visual cue, β-power was significantly increased in PD patients compared to healthy controls (Figure 7A).

### Oscillatory Brain Activity in Speech Production

Overt speech production is a highly complex task, accomplished by a distributed bilateral cortical and subcortical neural network (for a review, see Kemmerer, 2015). The brain areas that are crucial for the different stages of speech production are well known. Using functional magnetic resonance imaging (fMRI), activation was detected in the primary motor cortex, supplementary motor area, cingulate motor area, thalamus, basal ganglia, insula, temporal lobe and cerebellum during non-lexical speech production (Sörös et al., 2006). The information flow between these areas of the speech production network, in contrast, is only partially understood. Models of speech production propose detailed motor plans that activate dozens of muscles in a well-defined order on a millisecond time-scale and that are constantly updated by auditory and somatosensory feedback (Hickok, 2012).

Multiple lines of evidence, using electroencephalography, magnetoencephalography and electrocorticography, have demonstrated that electrical brain oscillations are crucial for information flow within distributed brain networks (Pfurtscheller and Lopes da Silva, 1999; Fries, 2005; Schnitzler and Gross, 2005; van Wijk et al., 2012; Cheyne, 2013). One of the most widely replicated finding is that preparation for and execution of limb movements are correlated with a gradual decrease of oscillatory power in the β-band, starting about 1 s before movement onset (Jasper and Penfield, 1949; Pfurtscheller, 1981; Leocani et al., 1997; for a review, see Hari and Salmelin, 1997; Cheyne, 2013; Kilavik et al., 2013).

Similar to voluntary limb movements, the preparation of speech production is also associated with a decrease of β-power (Gehrig et al., 2012; Jenson et al., 2014; Mersov et al., 2016). Mersov et al. (2016) studied brain rhythms of fluent speakers and adults who stutter using magnetoencephalography before and during cued overt reading of words in a carrier phrase. During speech preparation, a decrease of β-power (in this study: 15–25 Hz) was found in the bilateral cuneus of fluent individuals. In addition, a decrease of β-power was found in the mouth motor cortex (Mersov et al., 2016). Similarly, Gehrig et al. (2012) used magnetoencephalography to investigate the preparatory phase of overt and covert reading in healthy individuals. The authors found a decrease of β-power in the bilateral parietal lobe and the left articulatory motor region during preparation, beginning about 350 ms after the onset of a visual preparation cue (Gehrig et al., 2012).

Although the dynamics of β-power have been investigated for decades, the functional significance of event-related fluctuations of oscillatory brain rhythms has not been fully elucidated (Cheyne, 2013). The pre-movement decrease of β-power is thought to reflect the preparation for the motor response (Pfurtscheller and Lopes da Silva, 1999; Cheyne, 2013; Kilavik et al., 2013). In contrast, an increase of β-power in the motor cortex preserves the current motor state and inhibits the initiation of new motor plans (Neuper and Pfurtscheller, 2001; Engel and Fries, 2010). Several studies suggest a causal relationship between β-oscillations and motor functions (van Wijk et al., 2012). Stimulation of the subthalamic nucleus at 20 Hz slowed the development of maximal grip force in PD patients relative to healthy controls (Chen et al., 2011). Even in healthy adults, non-invasive transcranial alternating-current stimulation at 20 Hz over the contralateral motor cortex slows voluntary hand movements (Pogosyan et al., 2009).

### Brain Oscillations during the Preparation for Speech in PD Patients

With the advent of deep brain stimulation for the treatment of PD and the possibility to record local field potentials through the stimulation leads, an increase of β-band oscillations in cortico-basal ganglia circuits at rest (Brittain and Brown, 2014), even at an early stage of the disease (Pollok et al., 2012), has been described. Excessive β-band oscillations are believed to be directly linked to chronic dopamine depletion (Mallet et al., 2008) and are now regarded as neurophysiological signature of PD (Oswal et al., 2013). In PD patients who underwent bilateral implantation of deep brain stimulation electrodes in the subthalamic nucleus and who were off dopaminergic medication, a significant correlation was found between the power of local field potentials in a 8–35 Hz frequency band and clinical severity of PD as assessed by the total UPDRS III score (Neumann et al., 2016). Treatment with dopamine or deep brain stimulation of the subthalamic nucleus has been shown to decrease or even normalize excessive β-band oscillations (Giannicola et al., 2010; Jenkinson and Brown, 2011; Quinn et al., 2015). In PD patients off medication, the onset of pre-movement β-power decrease (Pollok et al., 2012; Meziane et al., 2015) was delayed during the preparation for limb movements. Heinrichs-Graham et al. (2014) used magnetoencephalography to study brain oscillations during planning, execution and termination of a tap of the right index finger in unmedicated PD patients and healthy age-matched controls. PD patients had a significantly smaller decrease of β-power prior and during movement than controls (Heinrichs-Graham et al., 2014).

While cortical and subcortical activity associated with limb movement has been widely investigated in PD, only a small number of studies have examined the neural correlates of speech production in PD. Functional neuroimaging research using positron emission tomography (PET; Pinto et al., 2004) and fMRI (Rektorova et al., 2007; Arnold et al., 2014) found increased activation, mainly in motor areas, in PD patients compared to controls. Overt speech production was associated with increased activation in the supplementary motor area (Pinto et al., 2004), primary orofacial sensorimotor cortex (Rektorova et al., 2007) as well as left dorsal premotor cortex and inferior frontal gyrus (Arnold et al., 2014) in patients vs. controls. For the study of brain oscillations, Hebb et al. (2012) recorded local field potentials in the subthalamic nucleus of PD patients during a continuous speech-language task, including naming the months of the year and counting upward from one. The authors observed a bilateral decrease of β-power preceding and during overt speech production in the subthalamic nucleus (Hebb et al., 2012). A recent study by Wojtecki et al. (2017) recorded subthalamic nucleus activity during a silent word generation paradigm, followed by overt pronunciation of the generated word. During silent word generation, an entirely cognitive task, the authors found a significant increase in α- and θ-power, but not in β- and γ-power. During overt speech, the recordings indicated a small, but insignificant decrease of β-power (Wojtecki et al., 2017).

To the best of our knowledge, an increase of β-power during the preparatory phase preceding movement has not been described before, neither in limb motor nor in speech control. The present study cannot provide definite answers regarding the functional significance of β-activity and its relationship to speech production, but may stimulate hypotheses for future research. Hyperactivity in speech motor areas, as demonstrated by PET and fMRI, has been interpreted as cortical mechanisms to compensate for basal ganglia dysfunctions. After all we know of the function of β-oscillations, an increase of β-power has an anti-kinetic effect (Jenkinson and Brown, 2011) and cannot be regarded as compensatory in nature.

It is important to note that the PD patients studied here were non-demented individuals with preserved verbal fluency. In addition, only fast, correct and intelligible responses have been included in the EEG analysis. Thus, the increase in β-power seen here does not necessarily cause speech production deficits. Moreover, the increase in β-power was seen in PD patients on medication, receiving a mean equivalent L-dopa dose of 677 mg/day. Importantly, the increase of β-band activity in our study was observed in the time window 200–400 ms after presentation of the visual cue, but not immediately preceding speech onset. Overt speech production is a multi-stage process that involves cognitive processes (mental retrieval of phonemes and combination of phonemes to syllables and words) and motor processes (developing an articulatory plan and execution of articulatory movements; Indefrey, 2011). Behavioral and electrophysiological studies delineated the temporal evolution of this cascade of events (Indefrey, 2011). The time window of the β-band increase observed here is dominated by a cognitive process, the retrieval of the phonological code. Planning of the articulatory movements starts later, about 150 ms before onset of articulation (Indefrey, 2011). This time course may explain why our patients had no symptoms of dysarthric speech with normal verbal fluency in the neuropsychological assessment and normal speech latency during the EEG recording. Our results would be compatible with an impairment of phonological processing, which has not been expected before and which was not tested explicitly in this study.

Our study has important limitations. In order to design a short experimental paradigm, feasible for patients with PD, we included only 25 trials per condition. In a future study, we would prefer 50 or more trials per condition to increase the signal-to-noise ratio of the EEG recordings. Moreover, a relatively high number of participants had to be excluded because of ongoing muscle artifacts. In a future study, we would take more time to familiarize the participants with the speech production paradigm and train them to relax their facial and jaw muscles. Regarding the analysis of EEG data, we decided to reference to the average activity of both mastoid electrodes. As the choice of reference may influence spectral power, the use of a neutral reference may have advantages (Yao, 2001; Yao et al., 2005).

In conclusion, the changes of oscillatory brain activity preceding limb movement and speech production differ in PD. While the physiological decrease of β-power preceding movement onset is delayed and smaller in PD patients off medication and normalizes under dopaminergic treatment, speech production is preceded by an increase of β-power in our patients who were on dopaminergic therapy compared to controls. This fundamental difference in rhythmic brain activity may contribute to the poor efficacy of dopamine and deep brain stimulation in PD speech disorder. Future research is warranted to determine the generators and the pathophysiological role of β-oscillations in PD speech disorder. First of all, a comparison of rhythmic brain activity between PD patients with and without speech impairment is needed to establish the relationship between behavioral speech performance and brain oscillations. Second, speech-related brain oscillations should be studied in patients on and off treatment (dopaminergic medication or deep brain stimulation) to gain further insights into the effects of antiparkinsonian treatment on speech-related β-power. Finally, a dedicated study on phonological processing in PD should find out whether the abnormal brain activity seen here influences cognitive processes in speech production and contributes to the pathophysiology of PD speech disorder. The results of these investigations may help to improve the currently often unsatisfactory therapy of this frequent and debilitating symptom of PD, e.g., through modification of deep brain stimulation protocols or further development of non-invasive stimulation techniques.

## Author Contributions

PS and TFM conceptualized and designed the study. CW and MA-K collected the data. ND, CW and PS analyzed the data. PS, ND and CW prepared the figures. PS wrote the manuscript. ND, CW, MA-K, NB and TFM interpreted the data, provided important feedback and revised the manuscript.

## Conflict of Interest Statement

The authors declare that the research was conducted in the absence of any commercial or financial relationships that could be construed as a potential conflict of interest.

## Footnotes

^1^www.neurobs.com

^2^web.audacityteam.org

^3^http://electro-cap.com

^4^http://compumedicsneuroscan.com

^5^http://sccn.ucsd.edu/eeglab

^6^http://www.fieldtriptoolbox.org

^7^https://www.ibm.com/analytics/us/en/technology/spss/
